# Self-Reported Comfort and Use of Tobacco Cessation Interventions by Healthcare Providers

**DOI:** 10.7759/cureus.74037

**Published:** 2024-11-19

**Authors:** Anjali Singh, Daniel J Berger, Sarahrose Jonik, Meghan E Robbins, Steven King, Jessica Yingst

**Affiliations:** 1 College of Medicine, Penn State University College of Medicine, Milton S. Hershey Medical Center, Hershey, USA; 2 Departments of Emergency Medicine and Internal Medicine, Virginia Commonwealth University Health System, Richmond, USA; 3 Department of Pediatrics, University of Connecticut School of Medicine, Farmington, USA; 4 Department of Internal Medicine, University of Michigan Health, Ann Arbor, USA; 5 Department of Public Health Sciences, Penn State University College of Medicine, Milton S. Hershey Medical Center, Hershey, USA

**Keywords:** nicotine replacement, smoking, smoking cessation, tobacco, tobacco cessation

## Abstract

Background

The effects of tobacco use create a significant burden on the American healthcare system. The U.S. Preventive Services Task Force (USPSTF) recommends a tobacco cessation framework consisting of asking all patients about any tobacco use, advising they quit, assessing their willingness to start a quit attempt, assisting in any attempts, and arranging follow-up. This is known as the “5A’s” and is considered a standard of care for tobacco cessation. Physician-provided cessation interventions have been shown to be effective in helping patients stop their tobacco use; however, studies have shown that physicians and other healthcare providers do not consistently offer tobacco cessation interventions. This study aimed to evaluate healthcare providers’ comfort with and self-reported use of tobacco cessation interventions.

Methods

An online survey was made available to all Penn State Health Milton S. Hershey Medical Center physicians, physician assistants (PAs), nurse practitioners (NPs), registered nurses (RNs), and respiratory therapists (RTs). The survey assessed respondents’ use of the USPSTF “5A’s” cessation framework, comfort in counseling patients, use of cessation interventions, and desire for further education. Descriptive statistics were generated, and chi-square tests were used to compare differences in responses across provider groups.

Results

A total of 430 healthcare professionals (mean age of 40.1 years, 76.1% female) responded to the survey, including 55 (12.1%) physicians, 76 (17.7%) resident/fellows, 44 (10.2%) PAs, 57 (13.5%) NPs, 146 (33.9%) RNs, and 54 (12.5%) RTs. The majority (n = 407, 95.5%) of respondents reported a belief that it is “extremely” or “very” important for their patients to stop smoking cigarettes. Although more than 160 (70%) providers reported feeling “very comfortable” or “somewhat comfortable” counseling patients who were “ready to quit” smoking, only half reported the same for patients who were “not ready to quit.” There was significant variation in the use of the recommended “5A’s,” with NPs and attending physicians reporting the most regular use. Self-reported use of the “Ask” and “Advise” components of the “5A’s” was higher than the “Assess”, “Assist”, and “Arrange” components, with low rates of use of pharmacologic cessation methods. Only 13 (3.2%) providers reported regularly billing for cessation counseling.

Conclusions

While healthcare professionals recognize the importance of tobacco cessation for their patients, gaps persist in the consistent application of the “5A’s” model and provider comfort in counseling patients to quit, particularly those perceived as “not ready to quit.” This discomfort with counseling, along with hesitancy to offer cessation interventions, results in missed opportunities to help patients with tobacco use disorder. Differences in cessation practices across healthcare roles suggest opportunities for targeted improvement. Enhancing both provider training and health system interventions is essential for expanding patient access to effective cessation interventions.

## Introduction

In the United States, tobacco use accounts for over 480,000 premature deaths annually and remains the leading cause of disease and mortality worldwide [1]. Unsurprisingly, the financial ramifications of tobacco use have resulted in healthcare expenditures upwards of $130 billion, with billions more spent as a result of lost productivity [2]. Additionally, there are numerous consequences associated with tobacco use, with varying levels of morbidity, including premature deaths from cancer (36%), heart disease and stroke (39%), and lung disease (24%) [2]. 

There are 83 carcinogens identified in cigarettes, some of which include polyaromatic hydrocarbons, n-nitrosamines, and aldehydes [3]. Tobacco use has been shown to cause cancers of the lung, oral cavity and pharynx, larynx, esophagus, stomach, colon and rectum, liver, pancreas, kidney, bladder, and cervix, as well as acute myeloid leukemia. Additionally, given the various harmful constituents of combustible tobacco smoke, tobacco users have an increased risk of developing abdominal aortic aneurysms (AAA), coronary artery disease (CAD), cerebrovascular accidents (CVAs), chronic obstructive pulmonary disease (COPD), pneumonia secondary to impaired immune function, poor asthma control, osteoporosis, and reproductive complications, among others [2].

Fortunately, a 22% global reduction in tobacco use by 2025 is expected [4]. This could, in part, be attributed to the increasing education and availability of both behavioral and pharmacologic interventions to assist with smoking cessation. For example, counseling as a form of behavioral intervention has been shown to be efficacious in promoting smoking cessation among tobacco users [5-7]. Additionally, nicotine replacement therapy (NRT), including nicotine patches or gum, has been shown to be effective in curbing nicotine addiction without the additive carcinogens in combustible tobacco smoke [5,8]. Pharmacologic agents like bupropion and varenicline are FDA-approved agents that are also available for smoking cessation. Lastly, there has been increasing data showing the efficacy of e-cigarettes as a method of smoking reduction/cessation, particularly as it has been gaining increasing popularity among tobacco users [5,8,9]. Evidence suggests that patients who utilize any method of cessation assistance are more than twice as likely to maintain a 12-month abstinence rate [5,10]. 

Given the negative effects of tobacco use and the numerous behavioral interventions and pharmacologic agents available for smoking cessation, it is important that healthcare providers address tobacco use with their patients. Currently, the U.S. Preventive Services Task Force (USPSTF) recommends that clinicians ask all their patients about tobacco use, advise them to quit, and provide behavioral interventions and appropriate pharmacotherapy [10]. This model, known as the “5A’s,” stands for “Ask, Advise, Assess, Assist, and Arrange” and can also be utilized through the “Ask-Advise-Refer” or “Ask-Advise-Connect” model [11]. Utilization of this model is considered a billable service by healthcare providers and has been shown to increase tobacco cessation by approximately two-thirds [12]. Current evidence suggests that patients are not routinely advised to stop their tobacco use or provided with the resources necessary to support their smoking cessation attempt [13,14]. Over the last several decades, attempts have been made in the hospital setting to increase documentation of both patient tobacco use and clinician assistance with tobacco cessation [15]. There is strong evidence that healthcare provider counseling is beneficial for patients struggling with cessation [5]. Although there has been an increase in health system documentation of patient tobacco use, it has not translated to changes in patient care [16]. 

Ultimately, current data surrounding healthcare provider knowledge and utilization of tobacco cessation efforts highlight an ongoing deficiency in the treatment of tobacco use disorder. For these reasons, we sought to assess the self-reported practices of healthcare providers regarding the use of the “5A’s” to initiate the cessation conversation with patients, along with their comfort with providing treatment, including counseling, referrals, and pharmacologic agents. This study additionally aimed to evaluate healthcare providers’ desire for additional education on tobacco reduction or cessation strategies across specialties and healthcare licensure.

## Materials and methods

An online survey was distributed to clinical staff at the Penn State Health Milton S. Hershey Medical Center, Hershey, PA, a 634-bed tertiary academic medical center, between September 2021 and December 2021. Surveys were publicized through an extensive recruitment campaign consisting of department emails, postings in health system newsletters, and flyers. The survey was open to all English-speaking healthcare providers, including physicians, physician assistants (PAs), nurse practitioners (NPs), registered nurses (RNs), and respiratory therapists (RTs) who were employed at Penn State Health Milton S. Hershey Medical Center. Participants who completed the survey were given the option to enter into a drawing to receive a $25 gift card. The survey was approved by the Pennsylvania State University Institutional Review Board (STUDY00020077). 

Study data were collected and managed using Research Electronic Data Capture (REDCap) tools hosted at the Penn State Health Milton S. Hershey Medical Center and the Pennsylvania State College of Medicine. REDCap is a secure, web-based application utilized to support data capture for research studies [17]. 

All participants received similar questions regarding the use of the “5A’s”, comfort in counseling patients, and willingness to provide cessation intervention. NPs and PAs (collectively referred to as advanced practice providers or APPs), along with physicians (collectively referred to along with APPs as providers), have both the ability to prescribe medications and to bill for cessation counseling. Therefore, they were asked about their willingness to provide specific interventions. In contrast, RNs and RTs were asked about their familiarity with specific interventions. The survey questions are available in Appendix 1. 

Statistical analysis 

Descriptive statistics were used to quantify responses, and chi-square tests were used to test for differences in proportions across different healthcare provider roles. Data were analyzed using SAS (SAS Institute, Cary, USA) version 9.4.

## Results

Four-hundred and thirty healthcare professionals responded to the survey, including 55 (12.1%) attending physicians, 76 (17.7%) resident/fellows, 44 (10.2%) PAs, 57 (13.5%) NPs, 146 (33.9%) RNs, and 54 (12.5%) RTs. They had a mean age of 40.9 years (SD = 13.1 years), and 76.4% were female.

Importance of tobacco cessation

Overall, 407 (95.5%) of responding healthcare professionals reported a belief that it is “extremely” or “very” important for their patients to stop smoking cigarettes, with significant differences across 62 (96.9%) attending physicians, 78 (97.5%) residents/fellows, 49 (98.0%) PAs, 64 (100.0%) NPs, and 154 (91.7%) RNs (p = 0.031). 

Use of the “5A’s”

**Figure 1:**
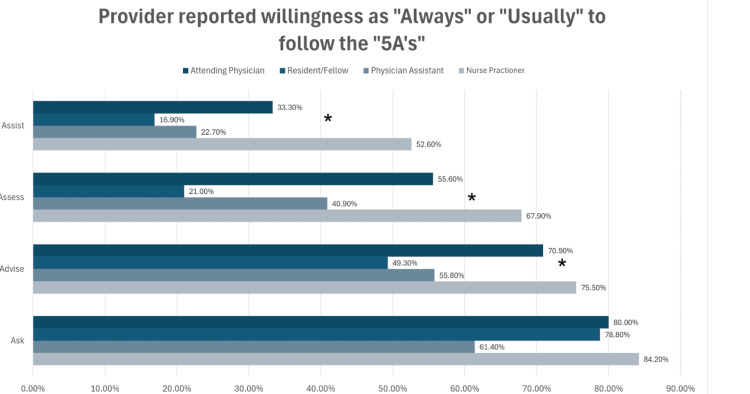
Self-reported regular use of the “5A's” Asterisks indicate significant differences (p < 0.05).

The reported use of the components of the “5A’s” varied significantly across professions. Among providers, NPs and attending physicians were more likely than PAs and residents/fellows to report “always” or “usually” using the “Ask, Advise, Assess, and Assist” components with their patients. Overall, there were significant differences in the use of the “Advise,” “Assess,” and “Assist” components across all providers, as shown in Figure 1 (p < 0.05).

Comfort counseling patients on tobacco cessation

Most respondents indicated comfort counseling patients who were “ready to quit,” with 58 (25.8%) providers reporting being “very comfortable” with counseling these patients and 106 (47.6%) reporting being “somewhat comfortable.” When disaggregated by license type, 58 (77.2%) residents/fellows, 57 (75.0%) NPs, 31 (69.8%) PAs, and 37 (70.4%) of attending physicians reported feeling “very comfortable” or “somewhat comfortable,” with no statistically significant difference across provider types (p = 0.127).

However, for patients who were perceived as “not ready to quit,” only 68 (16.9%) of providers reported being “very comfortable” and 173 (42.9%) “somewhat comfortable” offering cessation counseling, with significant differences (p < 0.05) seen across license types, 42 (55.7%) of residents/fellows, 41 (70.2%) of NPs, 25 (55.8%) of PAs, and 31 (60.0%) of attending physicians. 

Among non-providers, 43 (79.4%) RTs were “very” or “somewhat comfortable” counseling patients “ready to quit,” compared to 35 (65.5%) patients “not ready to quit.” This is compared to 43 (29.7%) and 10 (7.7%), respectively, for RNs.

Use of the specific interventions

**Figure 2:**
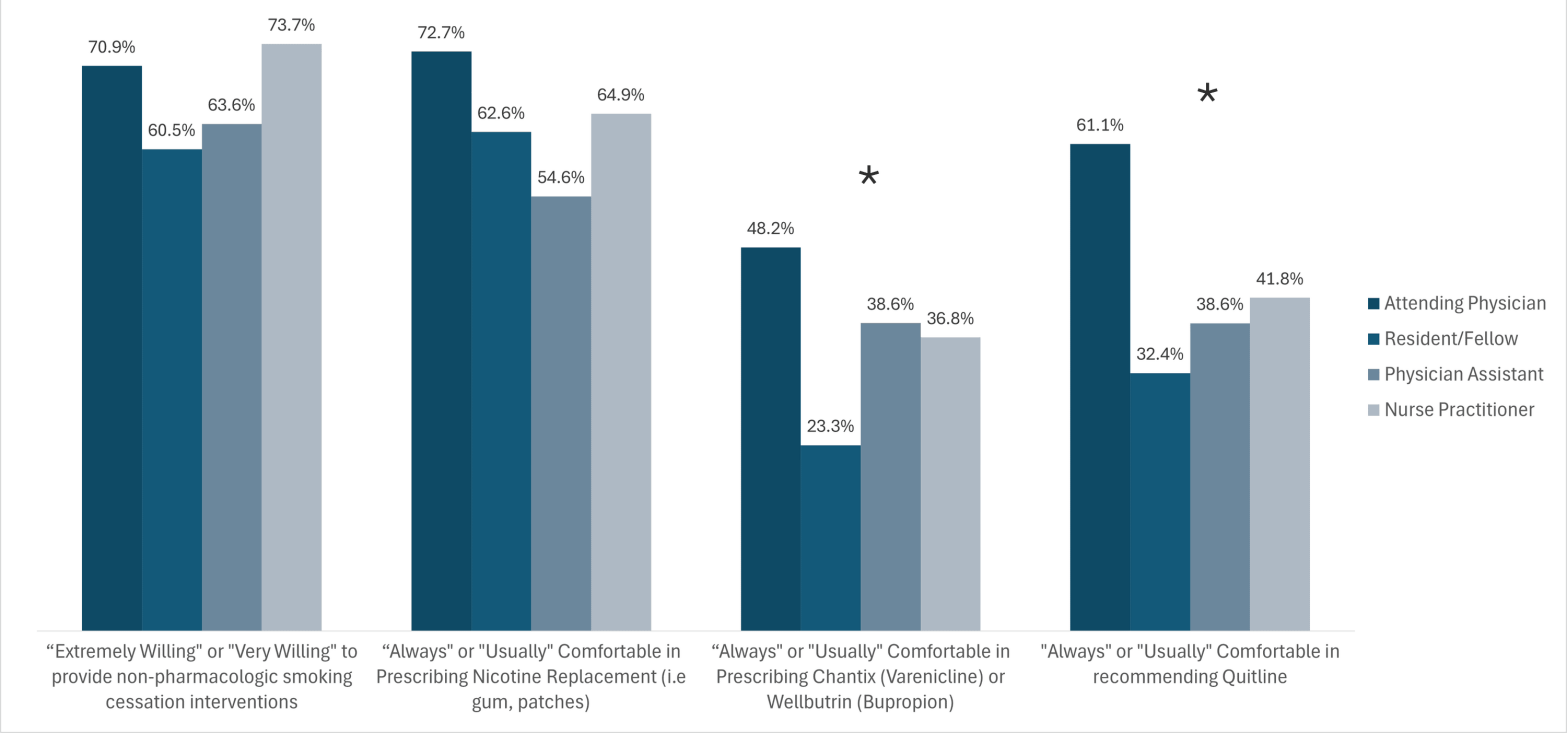
Self-reported provider comfort and willingness to use various cessation interventions Asterisks indicate significant differences (p < 0.05 across license types).

Willingness to use tobacco cessation interventions also varied between provider types, as shown in Figure 2. Among providers, 30 (52.6%) NPs reported that they “always” or “usually” use cessation intervention with their patients, compared to 17 (33.3%) attending physicians, 33 (22.4%) RNs, 10 (22.7%) PAs, and 13 (17.1%) residents/fellows.

Thirty-three (22.4%) RNs and 41.4% of RTs reported that they “always” or “usually” used cessation interventions with their patients. Eighty-six (58.6%) RNs and 37 (69%) RTs were “extremely” or “very” willing to offer non-pharmacological interventions.

Billing

Providers reported infrequent formal tobacco cessation counseling sessions lasting over three minutes, the minimum amount of time to qualify as a billable service. Formal counseling was “usually” or “always” done by 17 (29.8%) NPs, 14 (27.8%) attending physicians, eight (18.2%) PAs, and seven (8.6%) residents/fellows. Only 13 (3.2%) providers reported “usually” or “always” billing for formal counseling. Notably, no residents, fellows, or PAs reported that they “always” or “usually” bill for formal counseling.

Training on tobacco cessation

Overall, 110 (27.1%) respondents reported receiving previous training in tobacco cessation, but these varied across provider types, with 29 (47.5%) attending physicians, 37 (47.4%) residents/fellows, 14 (28.6%) PAs, 15 (24.2%) NPs, 15 (9.6%) RNs, and 30 (55.2%) RTs reporting previous training. Among those respondents who received training, the most common method reported was a lecture during professional school (n=87, 61.7%), followed by instructor-led live (in-person or virtual) sessions (n=66, 46.8%), self-directed online modules (n=42, 29.8%), and continuing education lectures (n=33, 23.4%).

## Discussion

This study found that although nearly all healthcare providers agreed on the need for smoking cessation treatment, only 72% of providers were comfortable counseling patients who were “ready to quit,” and just over half were comfortable counseling patients “not ready to quit.” These findings align with a study of psychiatrists, a field that includes substantial training on addiction, where only 70% were confident in their abilities to treat their patient’s tobacco use [18]. This underscores the need to increase provider comfort for initiating tobacco cessation, as this intervention has been associated with greater use of in-house cessation treatments and follow-up care [19]. 

The gap in comfort with counseling patients who were “ready to quit" compared to those who were “not ready to quit” is consequential, as providers report higher rates of provision of assistance and follow-up for tobacco users perceived as “ready to quit” [20]. This creates a potential disparity in care, as providers may be less likely to offer cessation interventions to those they perceive as “not ready to quit.” Unfortunately, studies have shown that young adult, Asian, American Indian/Alaskan Native, and Hispanic tobacco users are less likely to be advised by their healthcare provider to quit [21]. 

According to our study, attending physicians and NPs reported higher utilization of the “Ask” and “Advise” components of the “5A’s” method for tobacco cessation than residents/fellows and PAs. However, the use of the “Assess” and “Assist” components was lower across all provider groups. This is in line with previous surveys of providers finding high rates of use of the “Ask” (70-95%) and “Advise” (68-95%) components and lower use rates of “Assess” (61-85%), “Assist” (58-63%), and “Arrange” (1-23%) [18,20,22]. These findings were also supported by medical records, with one medical records study noting similar rates of screening (68%) but finding that less than 20% of patients were offered assistance, and less than 2% were prescribed pharmacotherapy [23].

In our study, RTs reported the highest level of comfort counseling patients perceived to be “ready to quit” and those “not ready to quit.” This is similar to a previous study that noted that general practitioners and RTs had the highest counseling scores for patients, regardless of their readiness to quit [20]. Notably, RNs reported lower levels of confidence than providers for both types of patients. This is consistent with previous literature, which also found high levels of comfort for RTs and lower counseling scores and “5A’s” utilization among RNs [20,22]. 

Lack of training may contribute to some of these differences in comfort and practice. In our study, only 25.2% of healthcare professionals reported receiving training in tobacco cessation interventions, with RTs and attending physicians being the most likely to have received training. Previous studies have shown that provider comfort and education are associated with higher rates of use of the “5A’s” and in-house cessation treatment [19,22]. An association between training and better cessation practices has also been documented with RTs [20,24]. Therefore, further education in smoking cessation interventions may be warranted for health professionals in their initial training and through continuing education. 

Additional education could be particularly important for RNs, especially those working in inpatient settings, who may have more patient-facing time to develop rapport with patients. RNs could be well placed to motivate their patients to quit, provide counseling, offer non-pharmacologic resources, and make connections to programs such as Quitline. However, our study demonstrated that they were the least likely to have received tobacco cessation training and were less comfortable counseling patients than other professionals. Previous studies have shown that RNs are less likely to use the “5A’s” [22] or offer follow-up [20] than other healthcare professionals. This may result from the type of education received, with nursing education often focusing on the health effects of smoking without emphasis on smoking cessation interventions [25]. 

In addition to comfort and previous education in tobacco cessation intervention, differences in perceived roles may affect willingness to provide tobacco cessation counseling. For example, RTs may have more direct exposure to the consequences of smoking, helping to reinforce this as a priority for their patients [24]. Similarly, Tong et al. found that primary care providers and psychiatrists were more likely than emergency medicine physicians and dentists to “assess,” “assist,” or “arrange” [22], so it is possible that some providers may not complete those steps if they do not view it as part of their professional responsibility.

A study of Italian cardiology and critical care nurses found that those who believed tobacco cessation was part of their clinical responsibility were more likely to report doing so [26]. This study also found that nurses cited time taken away from other nursing tasks as a reason why they did not perform tobacco cessation counseling, suggesting competing priorities may also act as a barrier. 

Our study found variation between providers in their reported use of pharmacologic and non-pharmacologic interventions, with attending physicians being more comfortable prescribing non-nicotine pharmacologic agents for smoking cessation, such as varenicline (Chantix) or bupropion (Wellbutrin, Zyban), compared to other provider types. However, all providers were willing to provide NRT and non-pharmacologic interventions. The combination of both counseling and medication is the current best practice for optimal smoking cessation among tobacco users [13]. Both sustained-release bupropion and varenicline have been considered first-line pharmacologic treatments for tobacco use since 2008 [6,15]. Of note, there was a nationwide Chantix recall in September 2021 that coincided with the start of data collection for this study. FDA-approved generic varenicline became available shortly thereafter, but the use of varenicline fell precipitously and was still lower than pre-recall rates in June 2022, without an increase in rates of bupropion use [27]. Potentially, the timing of the recall could have affected providers’ willingness to utilize pharmacologic interventions, though it would not explain the difference between attending physicians and other provider types. In the 2001-2004 National Ambulatory Care Survey, only 1.9% of tobacco users were offered pharmacologic intervention, which at the time consisted of bupropion [23]. There appears to be a long-standing trend of hesitancy toward the use of pharmacologic intervention, which is also evident in this study despite current evidence and clinical practice guidelines. 

Additional results corroborated this, as providers offered tobacco cessation counseling lasting longer than three minutes only 7.8-30% of the time, and less than 4% of all providers billed appropriately for these services. These findings align with previous studies that showed that health professionals’ interest in providing cessation support does not necessarily lead to continued follow-up [16]. The paucity of billing for tobacco cessation counseling could also reflect insufficient knowledge about the availability of reimbursement. In the United States, reimbursement can be a significant driver of practice changes, so increasing billing for tobacco cessation counseling could be investigated as a way to increase cessation interventions in concordance with present guidelines. The results of our study may reflect a lower rate of billing than would be expected in a non-academic institution, as physicians working in community settings often have their compensation tied to the services they bill for. Therefore, residents and academic faculty who may be compensated with a fixed salary may not see a direct benefit to billing for services.

In addition to financial incentives, billing for a service requires thorough and accurate documentation and offers a means to measure the quality of care [28]. Lack of formal education on billing in the resident curriculum can perpetuate inaccurate or insufficient billing [28]. Residents and fellows reported lower use of the “5A’s” compared to attending physicians and did not bill for tobacco cessation counseling services, which may reflect a lack of formal education as well as a lack of educator modeling. In addition, for formal curricula such as lectures, modeling by attending is a key way in which residents and fellows establish their practice patterns. Integrating tobacco cessation billing into auto-populated procedure code options would decrease barriers to billing for tobacco cessation counseling and serve as a visual reminder to offer it. Additionally, allowing billing for tobacco cessation counseling performed by RTs or RNs could also be explored as a method to expand access to tobacco cessation interventions. Both educational and system-based interventions may be considered in efforts to increase both the use of and billing for tobacco cessation counseling. 

For providers who are not willing to offer counseling or medication, the “Ask-Advise-Refer” or “Ask-Advise-Connect” format may be a viable alternative to connect patients with cessation resources and follow-up. For example, Westmaas et al. found practice differences in cessation interventions, with oncologists reporting higher use of referral to cessation services, while primary care physicians were more likely to offer cessation counseling themselves [19]. In our study, only 60% of attending physicians, 40% of APPs, and 30% of residents were comfortable referring patients to Quitlines, which has been shown to help patients quit smoking [12]. Our survey did not assess baseline knowledge of cessation resources, so it is not known if providers were truly uncomfortable referring patients to these services or if they were unaware of their existence or offerings. However, previous studies have also shown low use of such services and may provide an opportunity for additional education and health systems interventions to promote cessation referral [20,22]. 

System-level changes have been shown to improve providers’ use of cessation assistance for patients. A study by Drake et al. showed that the use of a clinical decision support intervention within the electronic medical record that pre-populated tobacco use in provider notes with documentation assistance to “nudge” providers to offer cessation interventions resulted in greater rates of successful quit attempts [29]. Self-directed interventions have also been suggested, such as integrating tobacco screening and cessation recommendations into tablet-based check-ins [30]. Ultimately, the Agency for Healthcare Research and Quality summarizes the importance of systems interventions, identifying “supportive systems, policies, environmental prompts, and insurance coverage” as necessary interventions to ensure consistent assessment and treatment of tobacco use [31]. 

Limitations

Limitations of this study include its single-site design, which could affect its generalizability as the institution may have a tobacco cessation “culture” that is different from health systems with more robust tobacco cessation programs. Additionally, the study population was a convenience sample asking about self-reported behaviors, so results may be affected by both non-response and recall bias. Furthermore, respondents’ data were not analyzed by specialty, and it is possible that some specialties with higher comfort with cessation counseling may have skewed results. The authors also created the survey, and the questions were not independently validated.

## Conclusions

Tobacco use remains the leading cause of preventable death, underscoring the critical role of all healthcare providers in providing tobacco cessation interventions to patients who use tobacco products as a standard component of medical care. This study demonstrates the ongoing need to enhance the implementation of evidence-based cessation interventions and reinforces the importance of ongoing education and training for healthcare providers. Equipping providers with the knowledge and skills to effectively address tobacco use and utilize FDA-approved interventions can significantly improve patient outcomes related to smoking cessation and ultimately reduce tobacco-related morbidity and mortality. Further integration of “5A’s” based on tobacco cessation interventions into healthcare provider training, use of health system-level interventions, and expanding the pool of providers allowed to provide tobacco cessation counseling as a billable service should be explored as methods to increase access to tobacco cessation for patients who use tobacco products.

## Appendices

**Table 1:** Tobacco cessation treatment questionnaire

Question	Answer choices
Please select your current position	Physician or advanced practice provider
	Registered nurse
	Respiratory therapist
	Student
	EMS
	Pharmacist
	Other
What type of clinician are you?	Attending physician
	Resident physician
	Fellow
	Physician assistant
	Nurse practitioner
How important do you believe it is for your patients to stop smoking cigarettes?	Extremely important
	Very important
	Somewhat important
	Slightly important
	Not important
Have you ever received smoking cessation training (course, formal lecture, etc.)?	Yes
	No
What type of training did you receive? Mark ALL THAT APPLY	Live session (instructor-led virtual or in-person)
	Online module (self-directed)
	Lecture during professional school
	CE lecture (after obtaining a license)
	Receive training FROM my healthcare provider (as a patient)
	Other
How often do you ask your patients about their tobacco use?	Never
	Rarely
	Sometimes
	Usually
	Always
For patients who report tobacco use or have it documented in their chart, how often do you: Explicitly ADVISE THEM to QUIT (e.g., "It is very important for your health that you stop smoking")	Never
	Rarely
	Sometimes
	Usually
	Always
For patients who report tobacco use or have it documented in their chart, how often do you: ASSESS their WILLINGNESS to QUIT (e.g., ask them if they are willing to start a quit attempt/set a quit date, want resources or medication, etc.)	Never
	Rarely
	Sometimes
	Usually
	Always
For patients who report tobacco use or have it documented in their chart, how often do you: Use any CESSATION INTERVENTION (QuitLine, furnish cessation medication, including nicotine replacement, motivational interviewing, provide literature, etc.)	Never
	Rarely
	Sometimes
	Usually
	Always
Please describe your comfort in your ability to offer smoking cessation COUNSELING to someone who is READY TO QUIT.	Very comfortable
	Somewhat comfortable
	Uncomfortable
	Very uncomfortable
Please describe your comfort in your ability to offer smoking cessation COUNSELING to someone who is NOT READY TO QUIT.	Very comfortable
	Somewhat comfortable
	Uncomfortable
	Very uncomfortable
What is your willingness to provide non-pharmacologic smoking cessation interventions? Interventions could include but are not limited to, counseling (>3 min), literature, and outside referral.	Extremely Willing
	Very willing
	Somewhat willing
	Slightly willing
	Not willing
How often do you OFFER formal smoking cessation to your patients (>3 minutes)?	Never
	Rarely
	Sometimes
	Usually
	Always
If you provide formal smoking cessation to your patients (>3 minutes), how often do you BILL for it?	Never
	Rarely
	Sometimes
	Usually
	Always
Please describe your COMFORT PRESCRIBING:	
Nicotine replacement (gum, patches, etc)	Very comfortable
	Somewhat comfortable
	Neutral
	Uncomfortable
	Very uncomfortable
Chantix (Varenicline) or Wellbutrin (Bupropion)	Very comfortable
	Somewhat comfortable
	Neutral
	Uncomfortable
	Very uncomfortable
QuitLine	Very comfortable
	Somewhat comfortable
	Neutral
	Uncomfortable
	Very uncomfortable
Smoking Cessation Clinic or Inpatient Consult Service	Very comfortable
	Somewhat comfortable
	Neutral
	Uncomfortable
	Very uncomfortable
Please describe your FAMILIARITY with the following:	
Nicotine replacement (gum, patches, etc.)	Very familiar
	Somewhat familiar
	Unfamiliar
	Very unfamiliar
Chantix (Varenicline) or Wellbutrin (Bupropion)	Very familiar
	Somewhat familiar
	Unfamiliar
	Very unfamiliar
QuitLine	Very familiar
	Somewhat familiar
	Unfamiliar
	Very unfamiliar
Smoking Cessation Clinic or Inpatient Consult Service	Very familiar
	Somewhat familiar
	Unfamiliar
	Very unfamiliar
